# Words and numbers: a comparative study of medical and journalism students’ descriptors of risk, numeracy and preferences for health risk communication

**DOI:** 10.1186/s12909-024-05048-3

**Published:** 2024-01-23

**Authors:** Eleanor Fallon, Norma Bargary, Fergal Quinn, Aisling Leavy, Ailish Hannigan

**Affiliations:** 1 Department of Mathematics and Statistics, University of Limerick, Limerick, Ireland; 2 Centre for Research Training in Foundations of Data Science, University of Limerick, Limerick, Ireland; 3 School of English, Irish and Communication, University of Limerick, Limerick, Ireland; 4 Department of STEM Education, Mary Immaculate College, Limerick, Ireland; 5 School of Medicine, University of Limerick, Limerick, Ireland; 6 Health Research Institute, University of Limerick, Limerick, Ireland

**Keywords:** Health, Risk communication, Education, Journalism, Medicine, Media

## Abstract

**Background:**

Given the complementary roles of health professionals and journalists in communicating health risks to patients and the public, there have been calls for physicians to work with journalists to improve the quality of health information received by the public. Understanding the preferences of medical and journalism students for the way in which health risks are communicated and their understanding of words used to describe risk is an important first step to inform interdisciplinary learning.

**Methods:**

Medical and journalism students (*n* = 203) completed an online survey where they were given qualitative descriptors of risk such as ‘a chance’, ‘probably’ and ‘unlikely’, and asked to assign a number that represents what the word means to them. Different formats of communicating risk (percentages, natural frequency and visual aids) were provided and students were asked to select and explain their preference. A thematic analysis of reasons was conducted. Numeracy and perceived mathematics ability were measured.

**Results:**

Numbers assigned to the descriptor ‘A chance’ had the highest variability for medical students. Numbers assigned to the descriptor ‘Probably’ had the highest variability for journalism students. Using visual aids was the most popular format for risk communication for both courses (56% of medical students and 40% of journalism students). Using percentages was twice as popular with journalism students compared to medical students (36% vs. 18%). Perceived mathematics ability was lower in students with a preference for natural frequencies and in journalism students, however performance on an objective numeracy scale was similar for all three formats (percentages, natural frequency and visual aids). Reasons for choosing a preferred format included good communication, eliciting a response, or learning style.

**Conclusions:**

Education on health risk communication for medical and journalism students should emphasize the need for qualitative descriptors of risk to be combined with the best available number. Students are already considering their role as future communicators of health risks and open to tailoring the mode of presentation to their audience. Further research is required on the design and evaluation of interdisciplinary workshops in health risk communication for medical and journalism students to maximise the opportunities for future inter-professional working.

**Supplementary Information:**

The online version contains supplementary material available at 10.1186/s12909-024-05048-3.

## Background

Risk communication is defined as “the open, two-way exchange of information and opinion about risk, leading to a better understanding of the risk in question, and promoting better decisions” [1, p.1]. Effective risk communication is increasingly important in patient-provider interactions given the rapid advances in medicine and the need for shared-decision making [2]. Low levels of numeracy in the general population and challenges interpreting numerical expressions of probability may make qualitative descriptors of risk using words such as ‘likely’ and ‘rare’ seem easier to use than numbers. However, these descriptors have been shown to be “elastic” concepts with different interpretations in different contexts [3]. Naik et al. concluded that for effective risk communication to patients and the public, qualitative descriptors of risk must be accompanied by numerical descriptors [1].

In a systematic review of 35 studies, Akl et al. evaluated the effects of using alternative statistical presentations of the same risks and risk reductions on understanding, perception, persuasiveness and behaviour of health professionals, policy makers, and consumers [4]. The statistical presentations of risk included a frequency e.g. 1 in 20 or a percentage e.g. 5%. ‘Natural’ frequencies are commonly used to present the risk of an event occurring, given that another event is already known to have occurred e.g. of the women with a positive mammography test, 1 in X will actually have breast cancer [5]. Akl et al. reported that the risk of a health outcome is better understood when it is presented as a natural frequency rather than a percentage for diagnostic and screening tests but that there was a lack of research on how these alternative presentations affect actual behavior. Zipkin et al.’s systematic review evaluated various methods of numerical and visual risk display across several risk scenarios and concluded that visual aids improved patients’ understanding and satisfaction [6].

Petrova et al. reported that physicians tailored risk communication for patients with low numeracy by using visual aids in the context of cancer screening [7]. However, physicians who themselves had low numeracy misunderstood the risks and unintentionally misled patients. The need to improve numeracy, risk literacy, and statistical skills training in medical curricula and continuing education was highlighted to ensure high-quality risk communication. This is important not just for patient-provider interactions but also for healthcare professionals communicating to the public in an era of health misinformation [8].

There is a growing body of evidence on the importance of information presented in the media in shaping patients’ perceptions of health risk. Suppli et al. used immunisation data, published news items and Google search activity to investigate the relationship between media coverage and HPV-vaccination acceptance in Denmark [9]. A changing point in correlation between media coverage and vaccination uptake was identified that coincided with an increase in Google searches for ‘HPV side effects’ and media coverage with negative content. It was suggested that negative media coverage may influence the decline of vaccination uptake. In an experimental study on the impact of different formats of news coverage of an epidemic on risk perceptions of the public, Klemm et al. found that perceptions of risk and severity were primarily driven by objective risk characteristics rather than emotion-laden news formats [10]. Journalists, therefore, can have a key role in presenting objective information about risk, yet often have no formal training in statistical reasoning [11] or presenting and interpreting medical research [12] and can rely on narratives rather than numbers [13].

Given the complementary roles of health professionals and journalists in communicating health risks to patients and the public, there have been calls for physicians to work with researchers and journalists to research best practices for communication and improve the quality of health information received by the public [8, 14]. Surveys of healthcare and media professionals have highlighted there can be a lack of trust and reluctance of healthcare professionals to engage with the media and have called for inter-professional training to address this [15]. Working together can develop shared understanding of both the health context and how media works [15], and promote evidence-based practice for communicating to the public [8].

To date, there has been no interdisciplinary comparative study of medical and journalism students’ preferences for health risk communication. Understanding the preferences of medical and journalism students for the way in which health risks are communicated and their understanding of words used to describe risk is an important first step to inform interdisciplinary learning and maximise opportunities for future inter-professional working. The aim of this exploratory study is to compare medical and journalism students’ understanding of qualitative descriptors of risk and their preferences for the format in which health risks are communicated. Specifically this study will address the questions:

- Are there differences in the numbers assigned by medical students and journalism students to qualitative descriptors of risk?
- What is the preferred format for communicating risk for medical students and journalism students?
- What are the reasons for choosing this format of presentation?
- Is choice of format associated with numeracy and perception of mathematics ability?
- Does preferred format influence decision-making about taking a drug?

## Methods

### Participants

All students in the pre-clinical years (Year 1 and 2) of a four-year graduate-entry medical degree (*N* = 286), a one-year Masters in Journalism (*N* = 19) and a four-year undergraduate degree in Journalism and New Media (*N* = 90), who were on campus in the autumn semester of the academic year 2019/20, were invited to participate in the study. Third year journalism students were off campus on work experience and were excluded. The medical degree includes a compulsory Biostatistics and Critical appraisal syllabus with three formal lectures on evaluating and comparing health risks. The amount of the syllabus covered at the time of this study varied by programme year (Year 1, 2). There is no formal teaching on health risks or health risk communication for students in the journalism programmes. These programmes can be characterised as broad-based, multi-media, skills-oriented degrees aimed at covering all journalism specialisations (i.e. TV, radio and written journalism) and do not contain medium or ‘beat’ (i.e. reporting specialisations such as science, economics or politics) specific pathways. This reflects typical approaches to journalism degrees in western university settings.

A member of the research team, not directly involved in the teaching of the students, presented information on the study to students at the end of a scheduled lecture and provided a web link to an online questionnaire. The information and web link were also emailed to all registered students. Participation in the study was voluntary and anonymous and all participants provided informed consent. Ethical approval for the study was granted by the Faculty of Education and Health Sciences Research Ethics Committee (reference number 2019_09_05_EHS).

### Measures

The survey instrument consisted of demographic information, and sections on expressions of risk and health risk communication. Six questions measuring numeracy were adapted from the expanded numeracy scale of Lipkus et al. [16]. The items were designed within the context of health risk and assessed the ability to (1) differentiate and perform simple mathematical operations on risk magnitudes, (2) convert percentages to proportions, and (3) convert proportions to percentages (see Supplementary Material 1). A single global attitude item on mathematics cognitive competence, adapted from the validated Survey of Attitudes Towards Statistics instrument [17] and tested previously in this population [18], was used to explore participants’ attitudes towards mathematics. Demographic information included age group, gender, country of birth, and native English speaker (yes, no). Information on course and year of study, any work experience relevant to their degree programme, and primary or postgraduate degree (for graduate-entry and Masters students) was also recorded.

#### Words and numbers describing risk

Participants were given the following illustrative examples of qualitative descriptors of risk:

1. This event is **unlikely** to occur.
2. This event will **probably** occur.
3. There is **a chance** this event will occur.
4. This event is **likely** to occur.
5. **I am confident** this event will occur.

and asked to select a number on a scale of 0 to 10 that represents what this word means to them where 0 is the event will definitely not happen, and 10 is the event definitely will happen.

#### Health risk communication

**Fig. 1:**
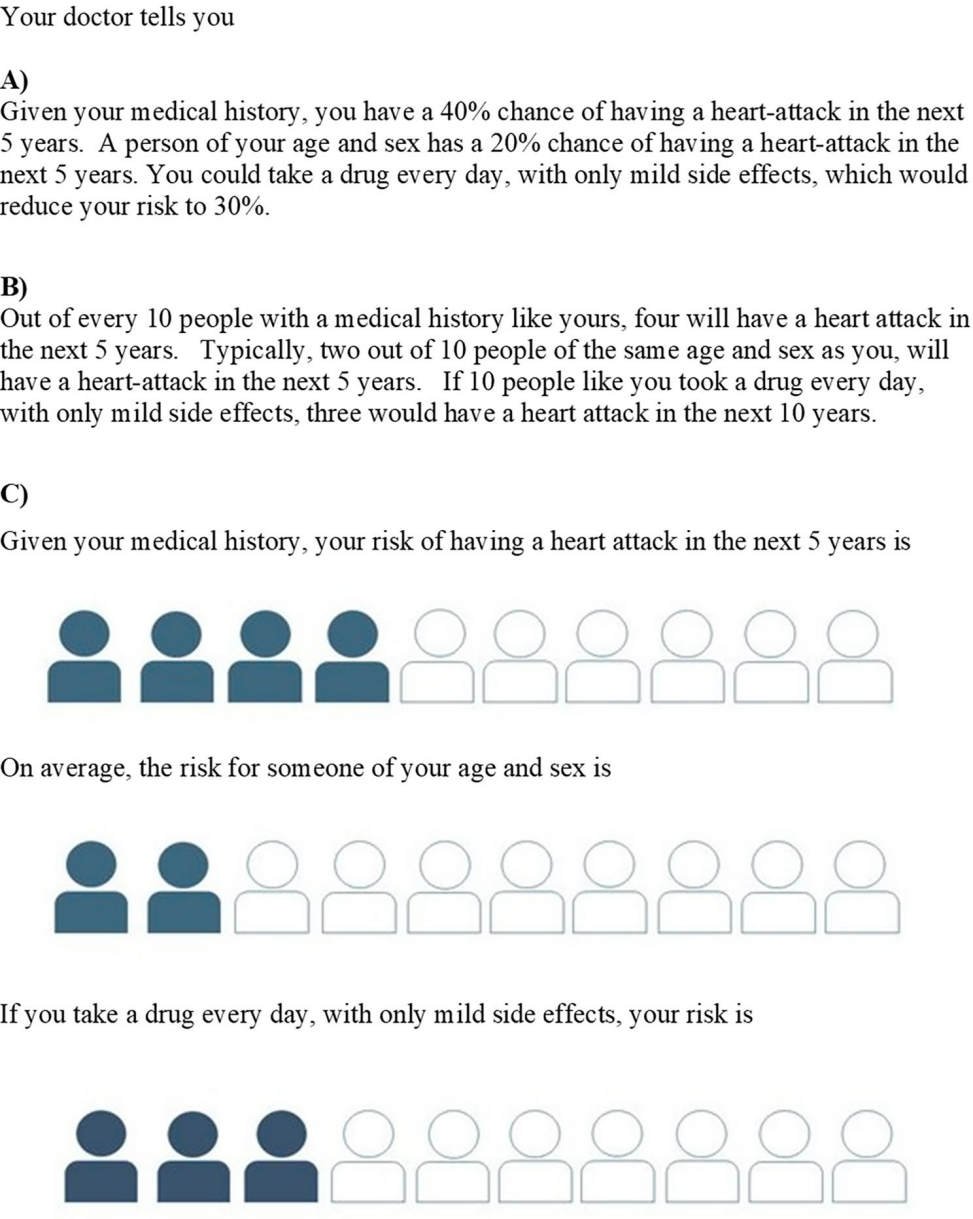
Different ways of communicating risk in a consultation between a patient and a doctor

As an illustrative example, three different formats of communicating risk in a consultation between a patient and a doctor were given to participants (Fig. 1). The choice of the example and the three formats (text with percentages, text with natural frequencies, and using icon arrays as visual aids) were informed by the literature on health risk communication [3, 4, 19, 20] and examples used in a large survey of the public on their perceptions of risk [21]. Participants were asked, firstly, which of the three formats they preferred and secondly, if they were the patient, would they take the drug. Participants were also provided with a free-text option to explain their preference for risk communication and their decision on whether to take the drug or not.

### Analysis

Categorical variables were summarised using counts and percentages. Numbers and ratings were summarised using the median and the interquartile range (first quartile, third quartile). An independent samples median test was used to compare median ratings of mathematical ability and numeracy across groups (medicine, journalism). A Mann-Whitney U test was used to compare distributions of numbers assigned to qualitative descriptors of risk across groups (medicine, journalism). A Kruskal-Wallis test was used to compare median numeracy across three groups (visual, natural frequency, percentages). A chi-square test was used to test for associations between categorical variables including would you take the drug (yes, no) and group (visual, natural frequency, percentages). A 5% level of significance was used for all tests. A sensitivity analysis was carried out on the results combining all journalism students into one group or split by the two journalism programmes (undergraduate, Masters).

A thematic analysis of free text comments on health risk communication preferences was carried out using a grounded theory approach. Three iterations of data analysis helped ensure the reliability of outputs [22]. As part of the first iteration of data analysis, one author looked for patterns and themes in participants’ responses. In the second iteration of data analysis, two other authors used these preliminary observed themes to independently analyse the data corpus and assess the explanatory fit of the elicited themes. The three researchers met to discuss the outcome of the second iteration of data analysis, to come to agreement on the dominant themes. The third iteration involved two researchers then using these themes to independently re-analyse the entire data set.

## Results

Of the 395 students invited to participate, 203 (51%) responded resulting in a response rate of 65% (71/109) for journalism students and 46% (132/286) for medical students. The characteristics of participants by course (medicine, journalism) are given in Table 1. A sensitivity analysis identified that results were broadly similar for all journalism students combined into one group or split into the two journalism programmes. Results are presented for the combined journalism group.

**Table 1:** Characteristics of participants (*n* = 203) by course

Characteristic	Medical students (*n* = 132) n (%)	Journalism students (*n* = 71) n (%)	Total (*n* = 203) n (%)
**Age group**			
18–21 years	4 (3%)	36 (50.7%)	40 (19.7%)
22–25 years	77 (58.3%)	24 (33.8%)	101 (49.8%)
26–29 years	31 (23.5%)	4 (5.6%)	35 (17.2%)
30 years or older	20 (15.2%)	7 (9.9%)	27 (13.3%)
**Gender**			
Male	47 (35.6%)	27 (38%)	74 (36.5%)
Female	85 (64.4%)	44 (62%)	129 (63.5%)
**Country of birth**			
Ireland	76 (57.6%)	52 (73.2%)	128 (63.1%)
Other European	8 (6.1%)	12 (16.9%)	20 (9.9%)
North America	36 (27.3%)	2 (2.8%)	38 (18.7%)
Other	12 (9.1%)	5 (7%)	17 (8.4%)
**Native English speaker**			
Yes	123 (93.9%)	57 (80.3%)	180 (89.1%)
No	8 (6.1%)	14 (19.7%)	22 (10.9%)
**Postgraduate qualification**			
Yes	39 (29.5%)	2 (2.8%)	41 (20.2%)
No	93 (70.5%)	69 (97.2%)	162 (79.8%)
**Relevant work experience**			
Yes	57 (43.2%)	23 (32.4%)	80 (39.4%)
No	75 (56.8%)	48 (67.6%)	123 (60.6%)

The majority of participants were female (*n* = 129, 64%), aged 25 years or younger (*n* = 141, 70%), born in Ireland (*n* = 128, 63%) and spoke English as a first language (*n* = 180, 89%). 20% of participants had a postgraduate qualification and 39% had at least one year of relevant work experience relevant to their degree programme. Medical students tended to be older, more likely to have a postgraduate qualification and less likely to be born in Ireland than journalism students, reflecting the graduate entry and international route into the medical programme. When asked ‘how good at mathematics are you on a scale of 1 to 7’, the median rating of perceived mathematics ability was lower for journalism students compared to medical students (median of 4 compared to 5, *p* < 0.001). Numeracy was measured by adding the total correct out of six questions on the adapted Lipkus numeracy scale, and scores were higher for medical students compared to journalism students (median of 6 correct vs. 5 correct, *p* < 0.001). The final two questions of the scale asked students to calculate with risks i.e. ‘double’ and ‘half’ numbers in different risk formats. Medical students performed better on these two questions compared to journalism students (89% correctly answered both questions vs. 51% respectively).

### Words and numbers describing risk

The numbers from 0 to 10 assigned by participants to qualitative descriptors of the risk of an event occurring are summarised in Table 2. Numbers assigned to the descriptor ‘A chance’ had the highest variability for medical students with 25% of medical students assigning it the number 3 or lower and 50% assigning it the number 5 or higher. Numbers assigned to the descriptor ‘Probably’ had the highest variability for journalism students with 25% of journalism students assigning it the number 5 or lower and 25% assigning it the number 8 or higher (Table 2).

**Table 2:** Median (first quartile, third quartile) from 0 to 10 assigned to qualitative descriptors of risk by course (*n* = 197)^1^

Qualitative descriptor	Medicine (*n* = 129)	Journalism (*n* = 68)	*p*-value^2^
Unlikely	2 (1,2)	2 (1, 3)	0.11
Probably	8 (7, 8)	7 (5, 8)	0.001
A chance	5 (3, 5)	5 (4, 6)	0.27
Likely	8 (7, 8)	8 (7, 8)	0.25
Confident	9 (9, 10)	9 (8, 9)	0.03

^1^ missing data for up to 8 participants

^2^*p*-value from Mann-Whitney U test comparing distributions

**Fig. 2:**
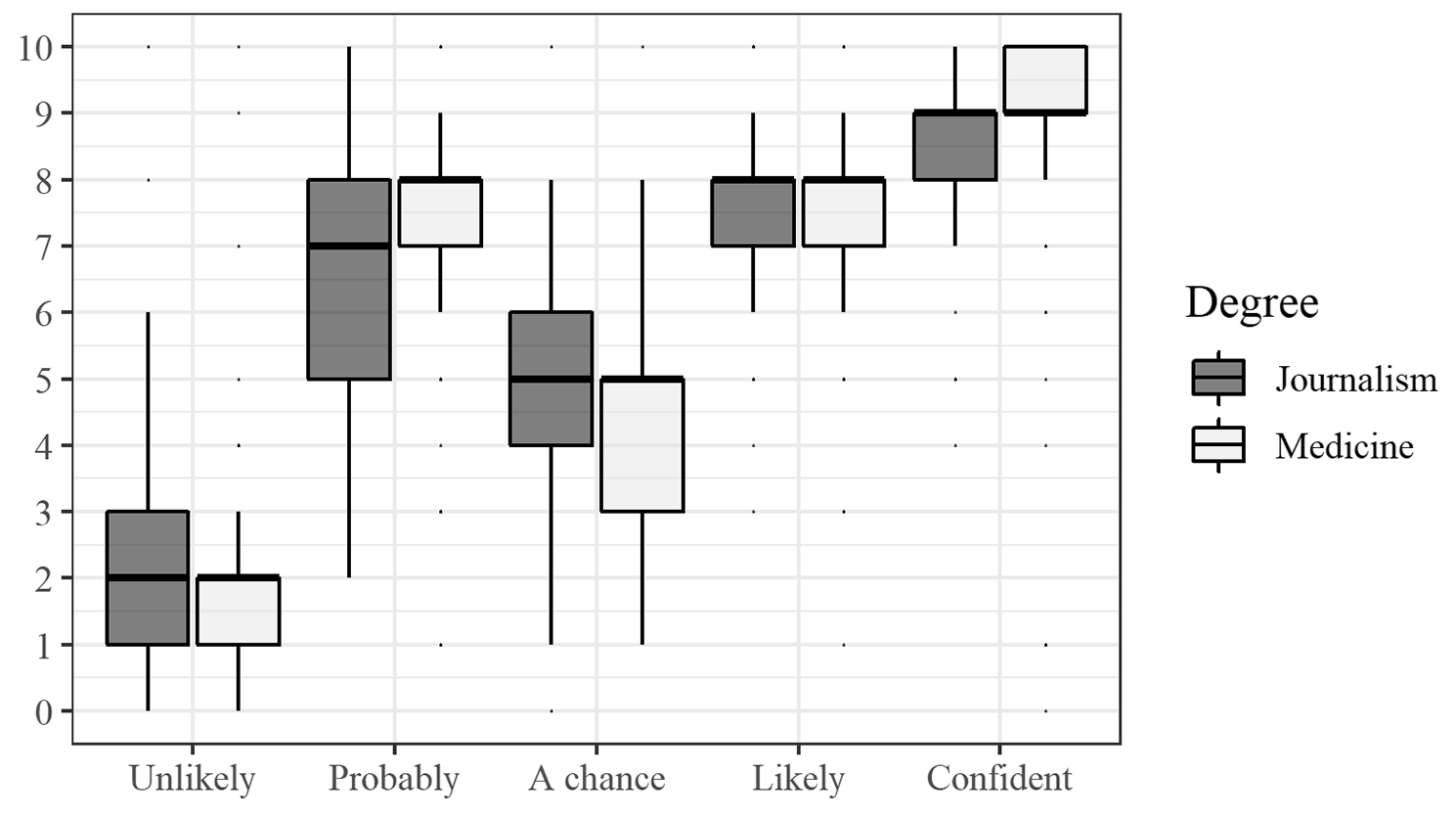
Distribution of numbers assigned to qualitative descriptors of risk by course. (*n* = 68 for journalism and *n* = 127 for medicine - missing data for 8 participants)

The distributions of numbers were similar across courses for the words ‘Unlikely’, ‘A chance’ and ‘Likely’ but different for the words ‘Probably’ and ‘Confident’. The wide variation in numbers assigned to ‘Probably’ is illustrated in Fig. 2 with a lower median value of 7 for journalism students compared to 8 for medical students. While the majority of students in both courses assigned the number 9 or 10 to being ‘Confident’ an event would occur, 25% of journalism students assigned the number 8 or below.

### Risk communication

**Fig. 3:**
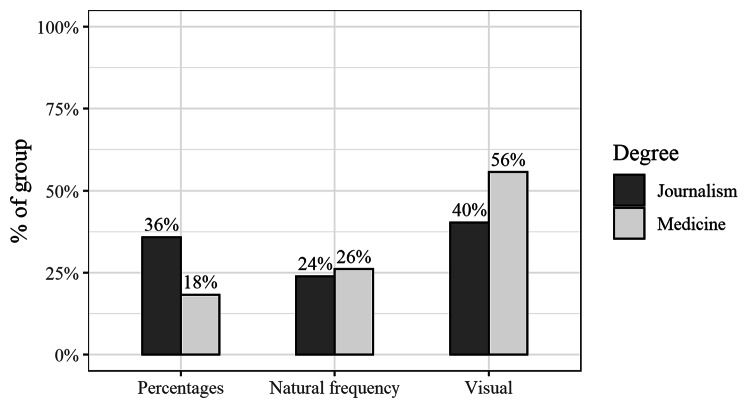
Preferred method for communicating risk by course (% of each course). (*n* = 67 for journalism and *n* = 115 for medicine - missing data for 21 participants)

The preferences for format of risk communication (percentages, natural frequency or visual) by course (medicine, journalism) are summarised in Fig. 3. The most popular preference for risk communication was using visual aids for both courses (56% of medical students and 40% of journalism students) but using percentages was twice as popular with journalism students compared to medical students (36% vs. 18%). Students who preferred the natural frequency format of communicating risk had a lower median of 4 on a scale of 1 to 7 for perceived mathematics ability compared to a median of 5 for students who preferred percentages or visual aids. Median scores for performance on the numeracy scale were similar for all three formats (median of 6 for each format, *p* = 0.63).

After deciding on their preferred format for communicating risk of a heart attack and how taking a drug would change that risk, participants were asked if they were the patient, would they take the drug? The majority of participants who responded to this question said yes (118/143, 83%) with high rates for both journalism students (88%) and medical students (79%). The percentage of those who would take the drug was 95% for those who preferred percentages as the format for communicating risk, compared to 76% for natural frequency and 81% for visual (*p* = 0.08). For those who decided not to take the drug (*n* = 25), the most common reason given related to a perceived insufficient reduction in risk to justify taking the drug (*n* = 11) or concerns/not enough information about side effects of the drug (*n* = 8).

### Reasoning behind risk communication preferences

Participants were asked why they chose their preferred format for communicating risk and 169 students (83% of participants) provided free-text comments. Three themes emerged: ‘Good Communication’, ‘Elicit a response’ and ‘Learning styles’. Themes, subthemes and examples of responses are given in Table 3.

**Table 3:** Themes, subthemes and examples of responses for choosing preferred method of communicating risk

Main themes	Subthemes	Examples of responses
Good communication	Easy to understand, straightforward Concise Precise, accurate, factual, direct Comprehensive Clear, obvious, stands out Simplicity Quick (fast) communication Engaging	“It was clearest and the percentages were easiest to understand” (M,P) “Concise, less, jargon” (J, NF) “more direct numbers” (J, P) “All the information was there and it was still clear to understand” (J, P) “Representation of the risk was clearer when presented as a number in every 10 people” (M, NF) “simpler, less complicated” (J, P) “Conveys the information quickly and succinctly” (M, V) “Visuals are always the best way to grab people’s attention” (J, V)
Learning style	Preference for visual over numerical style Preference for communication which is relatable Participant thinking of own learning style Participant thinking of how others learn	“I work better with visual aids as opposed to numbers” (J, V) “You can imagine 10 people and then the number of people who will get the disease” (M, NF) “I am a visual learner and tend to work better with visual representations” (M, V) “For individuals unfamiliar with risk and statistics I think depicting it in picture format would help improve understanding” (M, V)
Elicit a response	Provokes action Evokes emotional response	“It can help the patient to take action” (J, P) “When I hear 10 people I think of close friends and family” (M, NF)

M: Medical student; J: Journalism student; P: Percentages; NF: Natural frequency; V: Visual aids

The first theme, ‘Good communication’, focused on the quality and style of the description of risk in terms of how information was delivered. Subthemes that emerged included easy to understand, concise or quick, clear, simple and comprehensive. For example, a medical student chose percentages as the preferred method for communicating risk because “It was the clearest and the percentages were easier to understand” and a journalism student chose natural frequency because it was “concise, less jargon”.

The second theme that emerged was a preference for communication that acknowledged different ‘Learning styles’. Subthemes included preferences based on the learning style of the communicator themselves, learning styles of those who would receive the information, as well as preferences for modalities characterised as more ‘personal’. For example, a medical student chose visual as the preferred method for communicating risk because “I am a visual learner and can understand better with a visual representation” and a journalism student chose visual because it was “not boggy text with dense details”. A medical student who chose visual was considering learning styles of others and what was best to be “used clinically depending if a person is a visual or audio learner” and another “felt it was more personal to see human shapes rather than numbers”.

The third theme focussed on the ability of the method of communication to ‘Elicit a response’. Subthemes included provoking an action or specifically evoking an emotional response. For example, a journalism student chose percentages as the preferred method for communicating risk because “it can help the patient to take action” and a medical student chose natural frequency because “when I hear of 10 people, I think of close friends and family”.

We noted a difference between the themes that emerged for percentages and natural frequency and those that emerged for the choice of a visual method for communicating risk. Analysis of responses in this latter category saw ‘Learning styles’ feature far more prominently, particularly the learning style of those being communicated to. ‘Good communication’ was the dominant theme that emerged for both medical and journalism students who chose percentages or natural frequency as their preferred method for communicating risk. ‘Good communication’ in these instances tended to be characterised as ‘easy to understand’, ‘clear’ and ‘simple’. While the theme ‘Elicit a response’ features much less prominently in the thematic analysis than ‘Good communication’ and ‘Learning styles’ overall, responses from those who chose natural frequency as their preferred method for communicating risk were more likely to include the ‘evokes an emotional response’ subtheme.

## Discussion

This is the first interdisciplinary, comparative study of medical and journalism students comparing their own understanding of qualitative descriptors of risk and their preferences for health risk communication. We found considerable variation in the numbers assigned to qualitative descriptors both within and across medical and journalism students. The most variation for medical students was found for the descriptor ‘A chance’ of an event occurring. Differences across courses were particularly evident for the words ‘Probably’ (This event will probably occur) and ‘Confident’ (I am confident that this event will occur). The results of our study support the conclusions of Edwards et al. [2] that qualitative descriptors of risk are elastic concepts and the findings of Willems et al. who found large variability in the interpretation of laypeople and statisticians of probability phrases regularly used in Dutch news articles [23]. Willems et al. found more consensus in interpretations at the extreme ends of the probability phrases, for example ‘rare’ and ‘certain’, with less agreement for phrases such as ‘probable’ [23]. Our results support the need for raising awareness in medical and journalism students about this variability in the interpretation of qualitative descriptors of risk and the need to combine them with a number. This will enable objective risk communication that may, as suggested by Klemm et al., be the most important influencer of perceptions of risk from news reports [10]. These numbers may need qualifiers, however, which acknowledge that they are the best estimates available, given the current research evidence, and that estimates come with uncertainty [24].

The most popular format for communicating risk for both journalism and medical students was using visual aids and the dominant theme for choosing this format related to ‘Learning style’. Visual is one of the four sensory modalities identified by the Visual Auditory Read/Writing Kinesthetic (VARK) model of learning styles [25]. Visual learners prefer the depiction of information in diagrams, charts, graphs, and symbols. In contrast, Read/Writing learners prefer information displayed specifically as words in text. As well as their own learning style, students were also considering the learning styles of others and the best way of communicating to them, particularly those with language, literacy or numeracy barriers. Both journalism and medical students were therefore already considering their future role as communicators and tailoring the mode of presentation of information to their audience.

Journalism students were more than twice as likely to opt for percentages compared to medical students with a common theme in the reasons given of ‘Good communication’, particularly ‘easy to understand’. This may reflect percentages being familiar to journalism students who, unlike medical students in this study, have not had formal teaching in health risk communication where alternative methods such as natural frequency are presented. Percentages were the least favourite option of medical students. In a qualitative study of the presentation of side effects of medicines to consumers, consumers reported that natural frequencies were more ‘natural’ than percentages, required less computational processes and were easier to understand for small likelihoods than for example, a percentage of 0.01% [26]. Similarly, Oudhoff et al. reported that the ‘1 in X’ format was processed faster than other numerical formats [27]. The reasons given for choosing the natural frequency format of communicating risk tended to have the widest and most nuanced spread of themes and subthemes, especially among medical students. These included that natural frequency is easy to understand, clear, concise, likely to appeal to learning styles of both patients and media consumers, more personal generally and likely to evoke an emotional response.

Peters et al. studied the impact of format of presentation (frequency or percentages) on risk perceptions for people with different numeracy levels (high, low) [28]. Those with lower levels of numeracy perceived a side effect of a medication as less risky (on a scale from 1 = not risky to 5 = very risky) when given information in percentage format compared to frequency format, whereas the highly numerate gave similar risk perceptions in both formats. Barnes et al. found that women with lower numeracy were more likely to prefer graphical formats of communicating breast cancer risk compared to numeric risk formats [29]. In our study, students who opted for the natural frequency method of communicating risk rated their mathematics ability lower than students opting for percentages or visual aids. There was, however, no difference in objective numeracy scores across choice of format for the overall sample. In both groups of students, the majority would opt to take the drug and rates of students opting to take the drug were high for all three preferences of formats for communicating risk. For those not deciding to take the drug, a perceived insufficient reduction in risk and concern/not enough information about side effects of the drug were the most common reasons given.

Overall, the themes that emerged for choosing a particular format of presenting risk resonated with the findings in the literature on the impact of tailoring the mode of presentation of information to individual information modality preferences e.g. based on a preference for visual versus textual information. Students identified ‘Good communication’ which was clear and easy to understand, the ability to ‘Elicit a response’ and acknowledging different ‘Learning Styles’. The impact of mode tailoring identified in the literature includes satisfaction with the comprehensibility of information and positive effects on the evaluation, processing, and recall of information [30, 31]. The students in our study will practice in a world with increased access of patients to online health information and increased use of digital media in journalism. They are already considering how best to communicate to their audience and given that mode tailoring is a relatively simple and inexpensive tool for online information, further research is required on how to use it effectively in health risk communication by both physicians and journalists.

The COVID-19 pandemic highlighted the need for expert risk communicators and the need for trusted sources such as physicians to help fight mis- and disinformation [32, 33]. It also highlighted the key role of the media in the collection, aggregation and visualisation of data related to the outbreak [34] and the public risk it represented. Increasing the opportunities for collaboration and inter-professional learning with journalists could provide opportunities to promote evidence-based practice in health risk communication to patients and the public. These opportunities could include medical schools partnering with local journalism schools to embed training in the basics of journalism and health communication for medical students [14]. They could also include interdisciplinary workshops for both medical and journalism students where:

- motivations for the communication of health risks in different settings and contexts are discussed e.g. to individual patients or mass communication strategies, synchronously or asynchronously;
- the variability in interpretation of qualitative descriptors of risk is highlighted;
- the need to combine these descriptors with the best available number from published research is emphasized;
- preferences for different formats of risk communication are discussed with guidance on their appropriate use in different contexts.

Our findings can inform the design of these workshops to maximise opportunities for inter-professional learning and further research is required on their delivery and evaluation in both students and professionals. Limitations of our study include surveying students from one university only and the findings may not be generalizable. Further research could also replicate this study in other settings and contexts e.g. in other universities nationally and internationally, and in healthcare and media professionals.

## Conclusions

We found considerable variation in the numbers assigned to commonly used qualitative descriptors of risk within and across medical students and journalism students. This variation should be acknowledged and the need for these qualitative descriptors to be combined with the best available number should be emphasized in risk communication. The choice of format for presenting risk was influenced by rating of mathematics ability and course of study. The students in this study are already considering their role as future communicators of health risks and open to tailoring the mode of presentation to their audience, using different formats for presenting risk and acknowledging different learning styles. Further research is required on the design and evaluation of interdisciplinary workshops in health risk communication for medical and journalism students.

### Electronic supplementary material

Below is the link to the electronic supplementary material.

Supplementary Material 1

## Data Availability

The datasets generated and analysed during the current study are not publicly available under conditions of the Research Ethics Committee but are available from the corresponding author on reasonable request.

## References

[1] Naik G, Ahmed H, Edwards AG (2012). Communicating risk to patients and the public. Br J Gen Pract.

[2] Groene OR, Bergelt C, Ehrhardt M (2022). How good are medical students at communicating risk? An implementation study at three German medical schools. Patient Educ Couns.

[3] Edwards A, Elwyn G, Mulley A (2002). Explaining risks: turning numerical data into meaningful pictures. BMJ.

[4] Akl EA, Oxman AD, Herrin J, Vist GE, Terrenato I, Sperati F, Costiniuk C, Blank D, Schünemann H. Using alternative statistical formats for presenting risks and risk reductions. Cochrane Database of Systematic Reviews. 2011(3). 10.1002/14651858.CD006776.pub2 PMC6464912 21412897

[5] Gigerenzer G, Edwards A (2003). Simple tools for understanding risks: from innumeracy to insight. BMJ.

[6] Zipkin DA, Umscheid CA, Keating NL, Allen E, Aung K, Beyth R, Kaatz S, Mann DM, Sussman JB, Korenstein D, Schardt C (2014). Evidence-based risk communication: a systematic review. Ann Intern Med.

[7] Petrova D, Kostopoulou O, Delaney BC, Cokely ET, Garcia-Retamero R (2018). Strengths and gaps in physicians’ risk communication: a scenario study of the influence of numeracy on cancer screening communication. Med Decis Making.

[8] Krohn KM, Yu G, Lieber M, Barry M (2022). The Stanford Global Health Media Fellowship: training the next generation of physician communicators to fight health misinformation. Acad Med.

[9] Suppli CH, Hansen ND, Rasmussen M, Valentiner-Branth P, Krause TG, Mølbak K (2018). Decline in HPV-vaccination uptake in Denmark–the association between HPV-related media coverage and HPV-vaccination. BMC Public Health.

[10] Klemm C, Hartmann T, Das E (2019). Fear-mongering or fact-driven? Illuminating the interplay of objective risk and emotion-evoking form in the response to epidemic news. Health Commun.

[11] Dunwoody S, Griffin RJ (2013). Statistical reasoning in journalism education. Sci Communication.

[12] Copp T, Dakin T, Nickel B, Albarqouni L, Mannix L, McCaffery KJ, Barratt A, Moynihan R (2022). Interventions to improve media coverage of medical research: a codesigned feasibility and acceptability study with Australian journalists. BMJ open.

[13] Shaffer VA, Scherer LD, Focella ES, Hinnant A, Len-Ríos ME, Zikmund-Fisher BJ (2018). What is the story with narratives? How using narratives in journalism changes health behavior. Health Commun.

[14] Krohn KM, Crichlow R, McKinney ZJ, Tessier KM, Scheurer JM, Olson AP (2022). Introducing mass communications strategies to medical students: a novel short session for fourth-year students. Acad Med.

[15] Chapman B, Shankar R, Palmer J, Laugharne R (2017). Mental health professionals and media professionals: a survey of attitudes towards one another. J Mental Health.

[16] Lipkus IM, Samsa G, Rimer BK (2001). General performance on a numeracy scale among highly educated samples. Med Decis Making.

[17] Schau C, Stevens J, Dauphinee TL, Vecchio AD (1995). The development and validation of the survey of antitudes toward statistics. Educ Psychol Meas.

[18] Hannigan A, Hegarty AC, McGrath D (2014). Attitudes towards statistics of graduate entry medical students: the role of prior learning experiences. BMC Med Educ.

[19] Fagerlin A, Zikmund-Fisher BJ, Ubel PA (2011). Helping patients decide: ten steps to better risk communication. J Natl Cancer Inst.

[20] Galesic M, Garcia-Retamero R, Gigerenzer G (2009). Using icon arrays to communicate medical risks: overcoming low numeracy. Health Psychol.

[21] University of Cambridge. (2011). Science is a Risky Quiz-ness: a new study aims to assess how we perceive risk. https://www.cam.ac.uk/research/news/science-is-a-risky-quiz-ness-a-new-study-aims-to-assess-how-we-perceive-risks. Accessed 19 Dec 19.

[22] Woods P, Boyle M, Jeffrey B, Troman G (2000). A research team in ethnography. Int J Qualitative Stud Educ.

[23] Willems S, Albers C, Smeets I (2020). Variability in the interpretation of probability phrases used in Dutch news articles—a risk for miscommunication. J Sci Communication.

[24] Spiegelhalter D (2017). Risk and uncertainty communication. Annual Rev Stat Its Application.

[25] Fleming ND, Mills C (1992). Not another inventory, rather a catalyst for reflection. To Improve the Academy.

[26] Tong V, Raynor DK, Blalock SJ, Aslani P (2016). Exploring consumer opinions on the presentation of side-effects information in Australian Consumer Medicine Information leaflets. Health Expect.

[27] Oudhoff JP, Timmermans DR (2015). The effect of different graphical and numerical likelihood formats on perception of likelihood and choice. Med Decis Making.

[28] Peters E, Hart PS, Fraenkel L (2011). Informing patients: the influence of numeracy, framing, and format of side effect information on risk perceptions. Med Decis Making.

[29] Barnes AJ, Hanoch Y, Miron-Shatz T, Ozanne EM (2016). Tailoring risk communication to improve comprehension: do patient preferences help or hurt?. Health Psychol.

[30] Nguyen MH, Smets EM, Bol N, Loos EF, Van Weert JC (2018). How tailoring the mode of information presentation influences younger and older adults’ satisfaction with health websites. J Health Communication.

[31] Nguyen MH, van Weert JC, Bol N, Loos EF, Tytgat KM, van de Ven AW, Smets EM (2017). Tailoring the mode of information presentation: effects on younger and older adults’ attention and recall of online information. Hum Commun Res.

[32] O’Connor C, Murphy M (2020). Going viral: doctors must combat fake news in the fight against covid-19. Ir Med J.

[33] Bravo P, Martinez-Pereira A, Fernández-González L, Dois A (2023). What is needed to effectively communicate risk during a health crisis? A qualitative study with international experts based on the COVID-19 pandemic. BMJ open.

[34] Desai A, Nouvellet P, Bhatia S, Cori A, Lassmann B (2021). Data journalism and the COVID-19 pandemic: opportunities and challenges. Lancet Digit Health.

