# Supplementary material for: Words and numbers: a comparative study of medical and journalism students’ descriptors of risk, numeracy and preferences for health risk communication
Source: BMC Med Educ. 2024 Jan 23;24:84. doi: 10.1186/s12909-024-05048-3 (PMC10807177; doi:10.1186/s12909-024-05048-3)
Supplement: Supplementary file 1 — Supplementary Material 1 [file 12909_2024_5048_MOESM1_ESM.pdf]

1. If the chance of getting a disease is 10%, how many people would be expected to get the disease out of 1000?

[1] [10] [50] **[100]** [200]

2. If the chance of getting a disease is 20 in 100, this means that the % of people that get the disease is

[5%] [10%] [15%] **[20%]** [25%]

3. Which of the following numbers represents the biggest risk of getting a disease?

[1%] **[10%]** [5%]

4. Which of the following numbers represents the biggest risk of getting a disease?

[1 in 100] [1 in 1000] **[1 in 10]**

5. If person A's chance of getting a disease is 1 in 100 in 10 years and person B's risk is double that of A, what is B's % risk?

[1%] **[2%]** [5%] [10%] [20%]

6. 10% of people are expected to get a disease. A drug will halve this risk. If 100 people take the drug, how many would you expect to get the disease?

[1] [2] **[5]** [10] [20]
